# Experimental and Simulation Study of Solar-Powered Air-Gap Membrane Distillation Technology for Water Desalination

**DOI:** 10.3390/membranes13100821

**Published:** 2023-10-01

**Authors:** Mostafa AbdEl-Rady Abu-Zeid, Mohamed Bassyouni, Yasser Fouad, Toderaș Monica, Abdelfatah Marni Sandid, Yasser Elhenawy

**Affiliations:** 1 Department of Agricultural Engineering, Faculty of Agriculture, Suez Canal University, Ismailia 41522, Egypt; mostafa241981@agr.suez.edu.eg; 2 Center of Excellence in Membrane-Based Water Desalination Technology for Testing and Characterization (CEMTC), Port Said University, Port Said 42526, Egypt; 3 Department of Chemical Engineering, Faculty of Engineering, Port Said University, Port Said 42526, Egypt; 4 Department of Chemical Engineering, Faculty of Engineering, East Port Said University of Technology, North Sinai 45632, Egypt; 5 Department of Applied Mechanical Engineering, College of Applied Engineering, Muzahimiyah Branch, King Saud University, P.O. Box 800, Riyadh 11421, Saudi Arabia; yfouad@ksu.edu.sa; 6 Faculty of Sciences, University of Oradea, St. No.1., 410087 Oradea, Romania; 7 Mechanical Engineering Department, University of Ain-Temouchent, Ain-Temouchent 46000, Algeria; fateh.marnisandid@univ-temouchent.edu.dz; 8 School of Chemical and Metallurgical Engineering, University of the Witwatersrand, 1 Jan Smuts Avenue, Johannesburg 2000, South Africa; 9 Department of Mechanical Power Engineering, Faculty of Engineering, Port Said University, Port Said 42526, Egypt

**Keywords:** AGMD, polarization phenomena, Spearman’s correlation analysis, mathematical modeling, evacuated tube collector

## Abstract

This work aimed to investigate temperature polarization (TP) and concentration polarization (CP), which affect solar-powered air-gap membrane distillation (SP-AGMD) system performance under various operating conditions. A mathematical model for the SP-AGMD system using the experimental results was performed to calculate the temperature polarization coefficient (τ), interface temperature (T_fm_), and interface concentration (C_fm_) at various salt concentrations (C_f_), feed temperatures (T_f_), and flow rates (M_f_). The system of SP-AGMD was simulated using the TRNSYS program. An evacuated tube collector (ETC) with a 2.5 m^2^ surface area was utilized for solar water heating. Electrical powering of cooler and circulation water pumps in the SP-AGMD system was provided using a photovoltaic system. Data were subjected to one-way analysis of variance (ANOVA) and Spearman’s correlation analysis to test the significant impact of operating conditions and polarization phenomena at *p* < 0.05. Statistical analysis showed that M_f_ induced a highly significant difference in the productivity (P_r_) and heat-transfer (h_f_) coefficients (*p* < 0.001) and a significant difference in τ (*p* < 0.05). Great *F*-ratios showed that M_f_ is the most influential parameter. P_r_ was enhanced by 99% and 146%, with increasing T_f_ (60 °C) and M_f_ (12 L/h), respectively, at a stable salt concentration (C_f_) of 0.5% and a cooling temperature (T_c_) of 20 °C. Also, the temperature increased to 85 °C when solar radiation reached 1002 W/m^2^ during summer. The inlet heat temperature of AGMD increased to 73 °C, and the P_r_ reached 1.62 kg/(m^2^·h).

## 1. Introduction

Membrane distillation (MD) is a thermal separation process studied immensely in desalination and water treatment, wherein heat and mass are transported concurrently through the hydrophobic membrane matrix and pores [1]. The integration of ETCs and PV panels creates a synergistic effect. This means that the combined efficiency of the system can be greater than the sum of the individual components. For instance, surplus electricity generated by PV panels can enhance the system’s performance. Integrating ETCs and PV panels allows the SP-AGMD system to gather thermal energy (via ETCs) and electrical energy (via PV panels) from solar radiation. ETCs provide heat energy for the distillation process, crucial for separating water from impurities. PV panels generate electricity to power components like coolers and circulation water pumps, enhancing the self-sufficiency of the system. By utilizing solar energy, the SP-AGMD system reduces dependency on conventional energy sources, potentially leading to significant cost savings over time. The initial investment in ETCs and PV panels may be offset by long-term savings in energy costs, especially in regions with abundant sunlight. The reliance on solar energy minimizes the carbon footprint of the SP-AGMD system, making it more environmentally sustainable compared to systems powered by fossil fuels. Solar energy availability can fluctuate based on weather conditions and time of day. To ensure reliable operation, the system may incorporate energy-storage solutions like batteries or other backup power sources. The integrated system can be adapted to different climates and scaled up or down to meet varying energy demands or system sizes. In air-gap membrane distillation (AGMD), the difference in temperature between the hot and the cold feed solution creates vapor pressure differences across the membrane. Pure water can be extracted from saline water using an MD module at a hot inlet feed temperature that is lower than 100 °C [2]. Air-gap membrane distillation (AGMD) is one of the most efficient MD technologies due to the existence of the air-gap zone, which helps to increase conduction heat-transfer resistance across the membrane and decrease membrane fouling and wetting [3,4]. It has been stated that MD competition with other separation technologies depends on the generated permeation driving force, which is affected by feed temperature, salt concentration, flow rate, air-gap width, temperature polarization (TP), and concentration polarization (CP) [5,6].

### 1.1. Polarization Phenomenon

Temperature polarization occurs when the temperature at the membrane surface (T_fm_) is lower than the temperature of the feed solution (T_f_). This occurs due to conduction heat losses across membranes and convection through the air-filled membrane pores [7]. Concentration polarization occurs when the concentration of solutes at the membrane surface (C_fm_) is higher than the bulk concentration of the feed solution (C_f_). This arises from the accumulation of concentrated solutes near the membrane surface as water vapor is extracted.

### 1.2. Temperature Polarization (TP)

More than three decades ago, a theoretical investigation was first presented by Schofield et al. [8] on TP. Camacho et al. [9] and Alsaadi et al. [10] attributed low MD module productivity to TP and CP. More interestingly, Schofield et al. [11] conducted an experimental study on a direct contact membrane distillation (DCMD) module and proclaimed that TP reduces the vapor pressure at the hot membrane side and then module productivity. In this context, Curcio and Drioli [12] introduced a so-called temperature polarization coefficient (denoted by τ) to measure TP influence (i.e., thermal boundary layer thickness) on MD driving force and productivity. It was reported that the theoretical τ value approached the number 1.00 but practically changed between 0.20 and 0.90, according to the MD module used [13]. In experimental work, Phattaranawik and Jiraratananon [14] mentioned that the τ value of the DCMD system changed between 0.40 and 0.70. A value of 0.60 was reported for τ, which referred to a marginal TP effect, thus resulting in 40% productivity enhancement at an optimum feed inlet temperature of 60 °C. As described by Phattaranawik et al. [15], a comparison study was carried out between a spacer-filled channel and no spacer. The experimental results showed that the introduction of a spacer-filled channel significantly altered the thermal boundary thickness. It increased the τ value to 1.00 and subsequently enhanced the productivity of the DCMD module from 31% to 41%.

### 1.3. Concentration Polarization (CP)

Hwang et al. [16] investigated the influence of feed inlet temperature and velocity on DCMD module productivity (P_r_). It was reported that a significant improvement in productivity (P_r_) was found at higher feed velocities. Also, the mass transfer coefficient (k_f_) was changed from 0.0027 to 0.0042 L/(m^2^·h·Pa). The productivity of the module (Pr) declined as the NaCl concentration increased due to the increase in the concentration boundary layer thickness and reduction of the vapor pressure difference. Another DCMD system was established by Duong et al. [17] for the regeneration of LiCl solution. 

Other researchers [18,19] have conducted experimental studies to assess how feed salt concentration (C_f_) and concentration polarization (CP) affect MD productivity (P_r_). Results have shown that productivity (P_r_) dropped from 13% to 28% as C_f_ increased from 30 to 120 g/L, respectively. According to Martínez [20], the reduced MD productivity (P_r_) was attributed to a decrease in water activity and an increase in C_f_.

Janajreh et al. [21] stated that reducing the air-gap width in AGMD modules led to a 38% decrease in the τ value, indicating an increased thickness of the thermal boundary layer and resulted in an adverse effect on TP. In related research, Kurokawa et al. [22] tested the influence of acidic solution concentration and concentration polarization (CP) on the AGMD module productivity (P_r_). The authors reported that there was a significant decrease in module productivity (P_r_) attributed to the increase in the thickness of concentration boundary layers. The researchers showed that when compared to using pure water as a feed, the use of a NaCl solution decreased MD module productivity (P_r_) by approximately 40%. Furthermore, Calabro and Drioli [23] stated that a 4% productivity (P_r_) reduction was obtained when the NaCl solution was utilized as feed. Martínez and González [24] concluded that the impact of CP on water vapor pressure was comparatively less severe than that of TP, resulting in only a 0.2% decrease. Termpiyakul et al. [25] stated that when the MD module operated at low feed velocity, water characteristics such as high salt concentration and feed inlet temperature should be taken into account due to the prominence of TP. Also, Muhammad Suleman et. al. [26] reported that TP showed a greater influence on MD productivity (P_r_) in comparison to CP. Criscuoli [27] studied the effect of feed velocity on productivity (P_r_). It was stated that raising the velocity of feed created a turbulence flow regime near the membrane surface, which led to a remarkable change in the thickness of thermal boundary layers and a rise in the τ value. The impact of TP on the productivity (P_r_) of the vacuum membrane distillation (VMD) module was investigated experimentally by Alsaadi et al. [10]. The authors concluded that the sensitivity factor of module productivity to membrane mass transfer resistance and τ is inversely proportional to the operating parameters of feed bulk temperature and vacuum pressure. A comprehensive study was presented by Anvari et al. [28]. Innovative methods were reviewed to mitigate the detrimental effects of TP, such as advanced membranes (e.g., nano-structured surfaces, heated membranes under photothermal radiation, and metallic membranes), flow promoters (e.g., feed spacers, corrugated feed channels/membranes, and flashed feed channels), and self-heated MD systems (e.g., solar photothermal, joule, and induction heating). After reviewing several theoretical and practical studies in this area of research, it has become apparent that previous researchers primarily concentrated their efforts on assessing the influence of temperature polarization (TP) and concentration polarization (CP) on the performance of membrane distillation (MD) modules, as well as evaluating the performance of solar-powered membrane distillation (SP-MD) systems under various environmental conditions. To our knowledge, no prior studies have examined the combined impact of the MD process and solar energy on performance, particularly in relation to TP and CP. This represents a significant gap in the literature. Consequently, the current research endeavors to address this gap by investigating the synergistic effect of the air-gap membrane distillation (AGMD) process and solar energy on the performance of the SP-AGMD system, with a specific focus on TP and CP arising from water evaporation at the liquid–vapor interface at the hot-feed membrane. To achieve this, a meticulously designed SP-AGMD system was systematically analyzed under varying feed temperatures (T_f_), flow rates (M_f_), and salt concentrations (C_f_).

Solar-powered membrane distillation (SP-AGMD) was designed and investigated systematically under different feed temperatures (T_f_), flow rates (M_f_), and salt concentrations (C_f_). The experimental evaluation was implemented based on the computed values of productivity (P_r_), temperature polarization coefficient (τ), and heat-transfer coefficient (h_f_). It is difficult to measure the feed temperature (T_fm_) and concentration (C_fm_) experimentally at the membrane surface [9,29]. Thus, a mathematical model was introduced beneath for the AGMD module alongside the experimental data to determine each of T_fm_, C_fm_, h_f_, and τ.

Additionally, the AGMD module was simulated via the TRNSYS program using solar energy for validation. Therefore, an evacuated tube collector (ETC) was used for solar water heating. A photovoltaic (PV) system was used to supply the required electrical power for the cooler and circulation water pumps in the SP-AGMD system. The SP-AGMD system was studied under different climate conditions. The authors presented a quantitative characterization of the mass transfer process, and investigated the influence of TP and CP on the MD system performance by developing a descriptive mass and heat-transfer model. Simulation outcomes revealed that the TP and CP diminished the permeation driving force.

## 2. Mathematical Modeling of the SP-AGMD System

The mathematical model of SP-AGMD for a water desalination system is presented in two parts. The first part is the modeling of AGMD, and the second part is the simulation of thermal energy and electrical power sources for the desalination system driven by solar energy.

### 2.1. AGMD Mathematical Modeling

**Figure 1:**
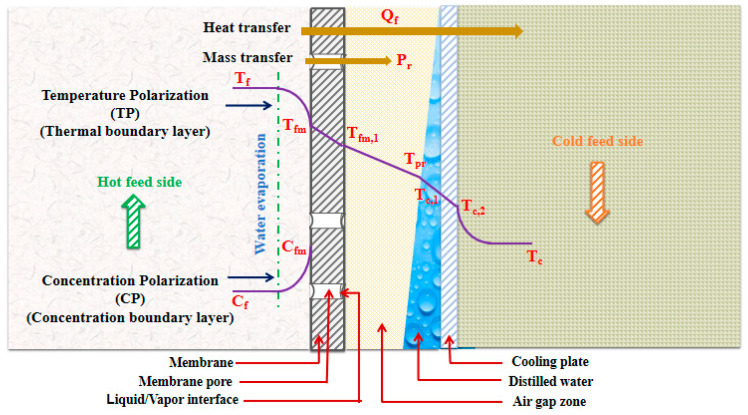
Temperature polarization (TP) and concentration polarization (CP) in the AGMD module.

A schematic diagram illustrating temperature polarization (TP) and concentration polarization (CP) taking place in the AGMD module is shown in Figure 1. At the liquid–vapor interface on the hot-feed membrane side where water evaporates, a simultaneous temperature decrease and concentration increase occur [30].

#### 2.1.1. Heat Transfer across the Thermal Boundary Layer Resistance

The thermal boundary layers on the hot-feed side impose further resistance to heat transport and render the feed bulk temperature (T_f_) larger than the interface temperature (T_fm_). This resulted in a reduction of 50% to 80% in driving force through the membrane. This phenomenon is defined as temperature polarization (TP) and could be recognized as the difference in temperature between feed bulk (T_f_) and membrane surface (T_fm_) [25,31]. The temperature polarization coefficient (τ) is defined as the ratio of the temperature drop across the membrane (from the hot-feed side to the cold permeate side) to the overall temperature difference between the hot-feed solution and the cold permeate solution. The TP influence can be determined by calculating the temperature polarization coefficient (τ) [8,32] using Equation (1).

(1) $$\tau \;=\;\frac{\;T_{fm\;}}{T_{f}}$$

In the case of T_fm_ < T_f_, a small τ value, high devastating TP impact, and unsatisfactory MD performance are the outcomes. On the contrary, when T_fm_ approaches T_f_, the τ is close to unity, implying a weak TP effect and better MD performance.

The effective convection heat transported (Q_f_) across the boundary layers can be determined using Equation (2).

(2) $$Q_{f}\;=\;\;h_{f}\;\times \;\;\left(T_{f}\;-\;\;T_{fm}\right)$$

where h_f_ is the heat-transfer coefficient through the tube-side thermal boundary layer (W/(m^2^·°C)). h_f_ could be estimated mathematically using Nusselt number (Nu) correlation, as given by Equation (3).

(3) $$Nu\;\;=\;\;a\;\;\times \;\;{\left(R_{e}\right)}^{b}\;\times \;{\left(P_{r}\right)}^{c}$$

where R_e_, P_r_, a, b, and c are the Reynolds number, Prandtl number, membrane modular design characteristic constants, and feed flow regime. R_e_ and P_r_ are given by Equation (4) [33].

(4) $$R_{e}\;=\;\frac{v\;\times \;d_{i}\;\times \;\rho }{\mu }\;\;P_{r}=\;\;\frac{c_{p}\;\times \;\mu }{k}$$

In the case of the current experimental operating conditions where a laminar flow regime (R_e_ < 2300; 0.6 < P_r_ < 5) through the lumen side has been exercised, Sieder and Tate’s equation could be applied according to previous studies [24,34] using Equations (5) and (6).

(5) Nu = 1.86 ∗  Re⁡ ∗ Pr⁡ ∗ diL0.33 

(6) $$h_{f}\;=\;\frac{N_{u}\;*\;k}{d_{i}}$$

where d_i_ is the internal diameter of the tube/hollow-fiber membrane (m), k is the liquid thermal conductivity (W/(m·°C)), C_p_ is the liquid heat capacity (J/(kg·°C)), µ is the bulk liquid dynamic viscosity (kg/(m·s)), ρ is the bulk liquid density (kg/m), and v is the linear velocity (m/s) that is calculated by Equation (7).

(7) $$\mathrm{Linear}\;\;\mathrm{velocity}\;(\nu )\;=\;\frac{F\mathrm{eed}\;\mathrm{flow}\;\mathrm{rate}\;(M_{f})}{\mathrm{Open}\;\;\mathrm{area}\;\;\mathrm{for}\;\;\mathrm{flow}\;\;\mathrm{through}\;\;\;\mathrm{the}\;\;\mathrm{tube}\;\;\mathrm{side}\;(A)}$$

#### 2.1.2. Heat Transfer across the Hollow-Fiber Membrane Pore

The distribution of heat transfer between latent heat and conduction heat loss depends on various factors, including the properties of the fluid, temperature gradients, material of the membrane, and overall setup. It is worth mentioning that these percentages can vary based on the specific conditions of the system, and the exact values might be influenced by the materials used, the geometry of the hollow fiber, the flow rate of the fluid, and the temperature difference across the membrane, among other factors. It was reported that 50% to 80% of latent heat (Q_v_) is lost across the dry pore, and 20% to 50% of the sensible heat (Q_c_) is lost [5,35]. The hot-feed solution evaporates at the membrane side. Then, vapor molecules diffuse through the pores as latent heat at a rate of Q_v_ = P_r_*∆H_v_, where P_r_ is the distilled water productivity, and ΔH_v_ is the evaporation latent heat (≈2326 kJ/kg).

The mean temperature of feed bulk (T_f_) was calculated using T_f_ = 0.5[T_fi_ + T_fo_]. The T_fm_ is hardly measured experimentally, but it could be determined theoretically via a simple enthalpy balance [8,36,37]:

(8) $$h_{f}\;*\;\left(T_{f}\;-\;T_{fm}\right)\;\;=\;\;\sum_{i=1}^{n}P_{r}\;*\;\Delta \;H_{V}$$

where n is the number of permeating species.

#### 2.1.3. Mass Transfer across the Concentration Boundary Layer Resistance

Increasing the salt concentration leads to an increase in the concentration boundary layer thickness (i.e., CP alongside the thermal boundary layers (i.e., TP)) and a decrease in driving force and, therefore, productivity [10,11,38]. The effect of CP is measured by calculating the concentration polarization coefficient (γ) using Equation (9) [38,39].

(9) $$\gamma \;=\;\frac{C_{fm\;}}{C_{f\;}}$$

where C_f_ and C_fm_ are the salt concentrations at the feed bulk and membrane surface, respectively. C_fm_ is estimated mathematically utilizing Equation (10) [6].

(10) $$C_{fm}\;=\;C_{f}\;*\;\exp\;\left(\frac{P_{r}}{\rho \;*\;K_{f}}\right)$$

where k_f_ is the solute diffusive mass transfer coefficient through the boundary layers (W/(m^2^·°C)). k_f_ can be computed using the Graetz–Lévêque Equation [40].

(11) $$k_{f}\;=\;\frac{Sh\;*\;D_{AB}}{d_{i}}$$

where D_AB_ and Sh are the diffusivity coefficient of water vapor (A) relative to air (B) in (m^2^/s) and Sherwood number, respectively. Sh could be determined through a laminar flow regime, as follows [41]:

(12) Sh = 1.86  ∗ Re⁡ ∗ Sc ∗  diL0.33

where Sc is the Schmidt number and can be computed by utilizing Equation (13) [6].

(13) $$Sc\;=\;\frac{\mu }{\rho \;*\;D_{AB}}\;$$

D_AB_ could be calculated mathematically at the feed bulk temperature varying from 273 K to 373 K using the Wilke–Chang empirical formula [42,43].

(14) $$D_{AB}\;=\;\frac{1.895\;\;*\;\;10^{-5}*\;\;T^{2.072}}{P}\;$$

### 2.2. Simulation Model of the SP-AGMD System

**Figure 2:**
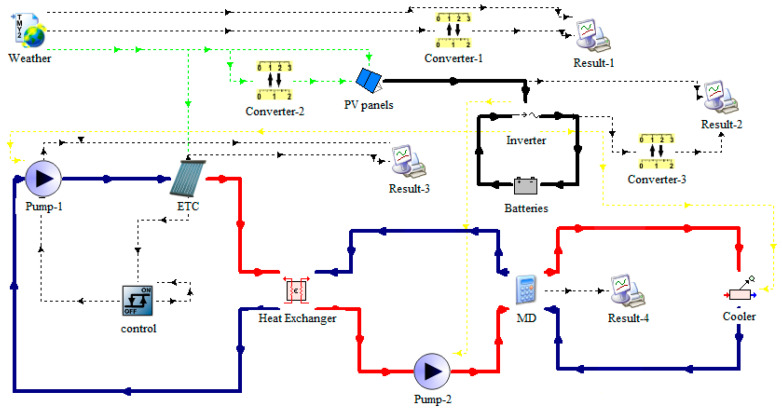
The SP-AGMD model uses a solar collector and photovoltaic panels.

The SP-AGMD model using a solar collector and photovoltaic (PV) panels was simulated by the TRNSYS program. As shown in Figure 2, the AGMD module was determined by a new equation in the TRNSYS simulation. All components of the solar AGMD model were presented as follows: a Type 91 heat exchanger, a TYPE109-TM2 reader and processer of meteorological data, a Type 2 differential temperature controller, a Type 1 flat plate collector, Type 94 photovoltaic panels, a Type 3 single speed pump, a Type 48 inverter, a Type 47 storage battery, Type 57 unit conversion, a Type 65 online plotter, and a Type 92 auxiliary cooling unit.

#### 2.2.1. Solar Thermal System

The thermostatic heating bath has an electrical power that reaches 1.5 KW. Therefore, the solar thermal system was simulated using solar energy for the AGMD system to save costs. The evacuated tube collector (ETC) was used for heating water in the AGMD system with an area of 2.5 m^2^ at coordinates 31°15′45″ N and 32°18′22″ E. In the solar thermal system, a heat exchanger has an effectiveness of 0.5. The parameters of ETC are listed in Table 1.

The basic method used to determine collector performance is given by Equation (15) [44].

(15) $${\dot{Q}}_{u}=m_{0}C_{pf}(T_{0}-T_{i})$$

where $m_{0}$ and $C_{pf}$ are the fluid mass flow rate (kg/h) and the specific heat capacity of fluid (KJ/h), respectively. $T_{0}\;and\;T_{i}$ are the exit and entrance temperatures of the collector (K).

The effectiveness of heat exchangers is given by Equation (16) [45].

(16) $$\varepsilon =\frac{1-\exp\left(-\frac{UA}{C_{min}}\left(1-\frac{C_{min}}{C_{max}}\right)\right)}{1-\left(\frac{C_{min}}{C_{max}}\right)\exp\left(-\frac{UA}{C_{min}}\left(1-\frac{C_{min}}{C_{max}}\right)\right)}$$

where UA is the overall loss coefficient among its surroundings during operation and the heater (kg/h). $C_{max}$ and $C_{min}\;$are the maximum and the minimum rate of heat capacity (KJ·hr^−1^·K), respectively.

#### 2.2.2. Photovoltaic (PV) System

The electrical power of the cooler circulation water pumps of ETC and AGMD is required for the AGMD process. Therefore, photovoltaic (PV) panels were used for the SP-AGMD system to save costs, as they constitute renewable energy. Therefore, the electrical power was calculated and replaced by two PV panels, each with an area of 1.6 m^2^ and a power of 300 W, using three batteries (12 V, 200 Ah) via the TRNSYS program. The power of the PV system covered the electrical power of the cooler circulation water pumps, according to the specifications listed in Table 2 [44].

The peak power of the PV installation is given by Equation (17) [44].

(17) $$P_{c}=P_{pv}=\frac{D}{N*F}$$

where D is the daily need in kWh/day, N is the number of hours, and F is the form factor, as given in Equations (18) and (19).

(18) $$N=\frac{G_{T}\left(t\right)}{G_{T,STC}}$$

where $G_{T}\left(t\right)$ is the solar radiation incident in the current time step on the solar PV array kW/m^2^. Under standard test conditions, $G_{T,STC}$ is the incident radiation kW/m^2^.

## 3. Materials and Methods

### 3.1. Experimental Setup Description of the SP-AGMD System

**Figure 3:**
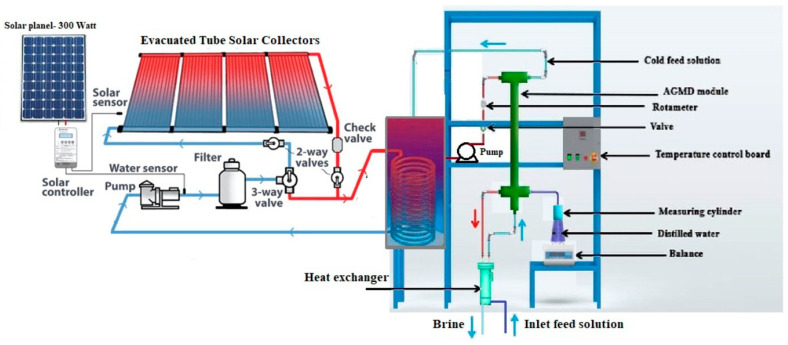
Schematic diagram of the experimental setup of the SP-AGMD system.

A schematic diagram of the SP-AGMD system utilized in this investigation is illustrated in Figure 3. The membrane distillation (MD) system consists of a feed tank, AGMD module, rotameter, water pump, electronic balance, measuring cylinder, valve, PV panels, evacuated tube solar collector, solar controller, and heat exchanger. The detailed specifications of the circulation pump, evacuated tube, and heat exchanger employed in this investigation are listed in Table 3. Electrical power to the water pumps was provided using solar panels. In the membrane distillation (MD) module, a feed solution was preheated using the heat exchanger and fed into the cold feed side. The outlet solution was heated using a solar heat exchanger to a specific temperature. A centrifugal pump was used to pump the outlet stream into the hot-feed side in AGMD. The evacuated tube collector (ETC) was used to provide the required heat to raise the temperature of the hot-feed side in the solar heat exchanger. Figure 3 schematically displays the itinerary of the salt solution inception from the feeding tank until it is collected as pure water in the measuring cylinder. The hot salt solution was pumped from the feeding tank into the PVDF membrane module by a circulation pump. After that, it crossed through the membrane module (indicated in red), where vapor diffused through the pores of the membrane.

On the opposite side, the hot solution exited the membrane module and fed into the cooler to reduce its temperature. Subsequently, it flowed through the PP heat-exchange tubes (indicated in blue) and was then cycled back to the feeding tank.

To maintain consistent water levels and feed concentration during the experiment, distilled water was introduced into the tank. To establish a stable operating state, the AGMD system was operated for an hour to eliminate all dissolved gases from the feed solution before commencing the experiment.

The changes, either increasing or decreasing, take place in the permeation driving force and AGMD productivity (P_r_), which is related to temperature polarization (TP) and concentration polarization (CP) under varying operating conditions. They are measured by estimating the temperature polarization coefficient (τ) and concentration polarization coefficient (γ), respectively. A temperature controller XMTD-3001 (Easey Commercial Building Hennessy Road Wanchai Hongkong, China) and thermostatic heating bath were installed to regulate the feed inlet temperatures (T_f_) at 50, 60, 70, and 80 °C. A cooler was used to maintain the temperature on the permeate side at a stable cooling water temperature (T_c_) of 20 °C. Four different temperature sensors were placed at the inlets and outlets of the membrane module to measure the variations that occurred in the feed temperature during operation. A rotameter was utilized to adjust the inlet flow rate (M_f_) at 3, 6, 9, and 12 L/h (equivalent to crossflow velocities of 0.014, 0.028, 0.041, and 0.055 m/s, and Reynolds numbers (Re) of 11.18, 22.36, 32.73, and 43.91, respectively). The productivity (P_r_) in kg/(m^2^·h) was calculated according to Aryapratama et al. [45] using Equation (19):

(19) $$P_{w}\;=\;\frac{W_{r}}{A\;\times \;t}$$

where W_r_ is the pure water volume (L), t is the experiment duration (h), and A is the effective membrane area based on the inner hollow-fiber membrane diameter (m^2^). The experiment was repeated three times for 1 h each under the same conditions, and the average of multiple values was calculated for accuracy. A conductivity meter (Model: DDS-11A, Shanghai Leici Xinjing Instrument Company) was used to measure the electrical conductivity of distilled and salt water (0.5%, 0.9%, 1.8%, and 4%) to check for any membrane pore wetting. The salt rejection rate (R_s_) was determined according to Li et al. [46], as given by Equation (20).

(20) $$R_{s}\;=\;\frac{C_{f}\;-\;C_{w}}{C_{f}}\;\times \;100$$

where C_f_ and C_w_ are the concentrations of salt and distilled water (%).

### 3.2. Air-Gap Membrane Distillation (AGMD) Module

The fabricated AGMD module contains a membrane made up of 120 porous polyvinylidene difluoride (PVDF) hollow fibers, and 240 non-porous polypropylene (PP) heat-exchange tubes with 0.36 m^2^ total interior membrane surface area. The interior/exterior diameter (m × 10^−3^) of the hollow-fiber membrane and heat-exchange tubes are 0.80/1.10 and 0.40/0.50, respectively. The length of the membrane and tube is 0.59 m. Polyvinylidene difluoride membrane thickness is 150 μm, the pore size is 0.20 µm, the contact angle is 80.5°, the bubble point pressure is 0.11 MPa, and the porosity is 85%. The average thickness of the air gap is 5 mm. The membrane module was insulated to avoid heat loss to the surroundings.

## 4. Statistical Analysis

Experimental results were statistically determined in terms of means and standard error for means (SE). One-way analysis of variance (ANOVA) was used to determine the effect of various operating conditions at *p* < 0.05. Additionally, the correlation coefficient (r) between the independent (i.e., feed temperature, flow rate, and salt concentration) and dependent variables (i.e., productivity (P_r_), temperature polarization coefficient (τ), and heat-transfer coefficient (h_f_)) was studied using Spearman’s correlation analysis. All statistical analyses were performed using IBM-SPSS version 23.0 for Mac OS [47,48].

## 5. Results and Discussion

It is worth mentioning that the low value of temperature polarization coefficient (τ) (i.e., increase in the thermal boundary layer thickness at the membrane surface) denotes the negative influence of temperature polarization (TP) on the productivity (P_r_) of the SP-AGMD system.

### 5.1. Effect of Temperature Polarization and Concentration Polarization on the Productivity at Different Feed Temperatures (T_f_)

**Figure 4:**
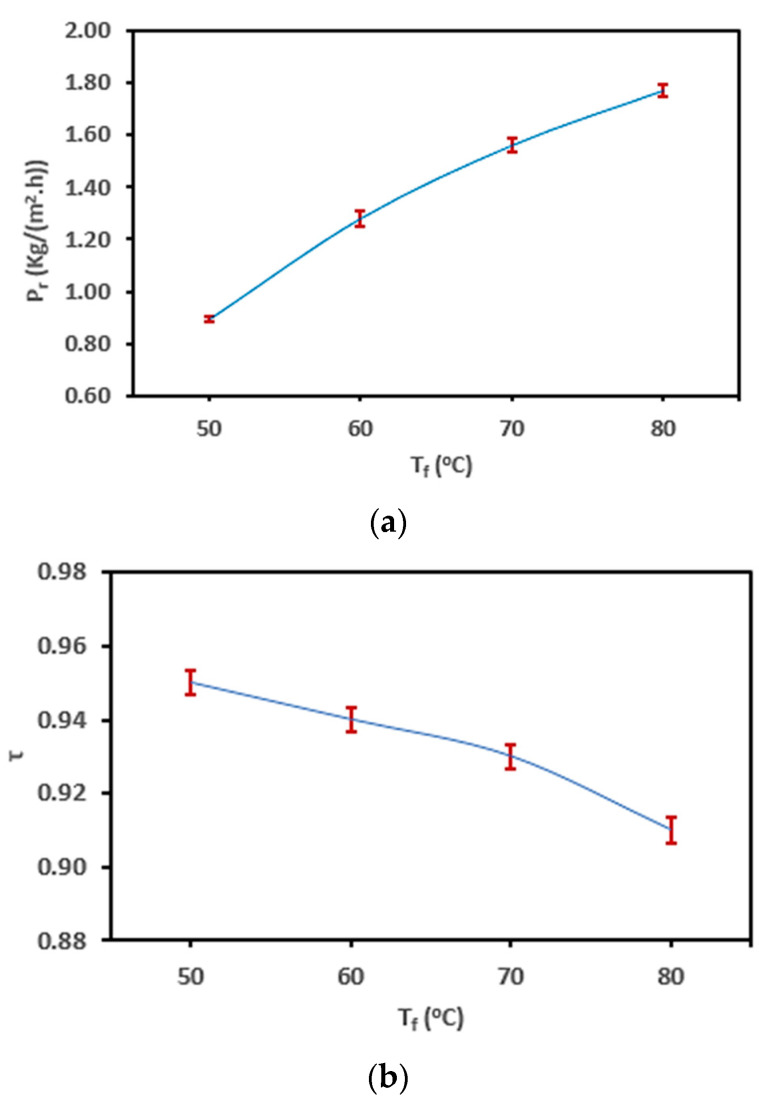

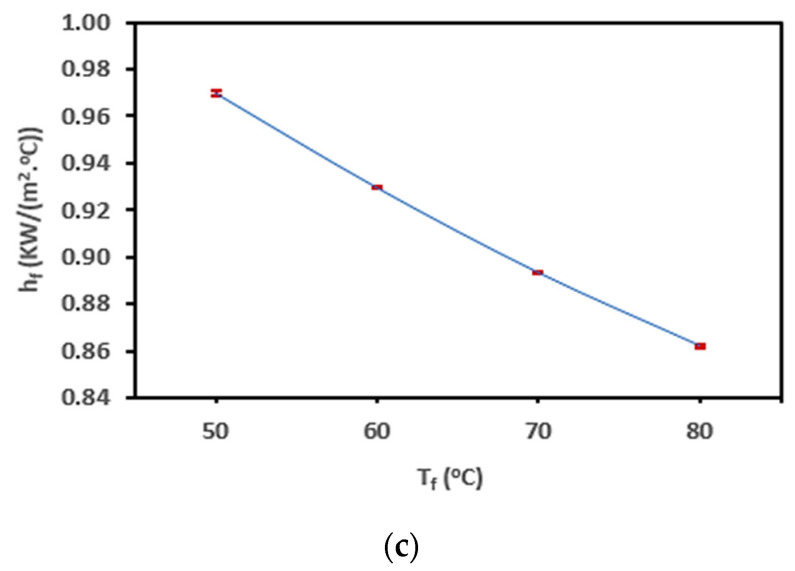
Change in the (**a**) P_r_, (**b**) τ, and (**c**) h_f_ for the SP-AGMD system at different feed temperatures (T_f_), with standard errors.

The variation of P_r_, τ, and h_f_ for the SP-AGMD system are elaborated in Figure 4a–c. Feed temperature (T_f_) changed between 50 °C and 80 °C at 10 °C intervals, and salt concentration (C_f_), coolant temperature (T_c_), and flow rate (M_f_) were kept at 0.5%, 20 °C, and 12 L/h, respectively. Exponential productivity (P_r_) increments with feed temperature taking place in the AGMD module were ascribed mostly to the corresponding exponential vapor pressure augmentation. In Figure 4a, the productivity (P_r_) of the AGMD module was enhanced from 0.89 to 1.77 kg/(m^2^·h) by 99% when the feed temperature (T_f_) was raised from 50 °C to 80 °C in increments of 10 °C. The improved productivity (P_r_) is attributed to the increase in vapor pressure and permeation driving force across the membrane, as predicted by the Antoine equation [35,49]. The higher temperature causes the liquid on one side of the membrane (the feed side) to evaporate more readily, generating a higher concentration of vapor molecules. This concentration difference across the membrane drives the transfer of vapor through the membrane to the other side, where it condenses and forms the purified product. The substantial increase in productivity (P_r_) (99%) shows the significance of temperature in AGMD processes. However, it is important to consider that changes in temperature might also influence other factors like energy consumption, membrane properties, and system stability. Therefore, while higher temperatures can enhance productivity, there might be practical limitations.

Related to the temperature polarization coefficient (τ), Figure 4b shows that τ declined by 2.0%, 1.80%, and 2.2% as the temperature (T_f_) increased. From these observations, it was found that the changes in temperature had a relatively minor effect on the temperature polarization coefficient (τ). A decrease in τ could potentially indicate a change in the temperature difference between the feed and permeate sides of the membrane, which could influence the driving force for vapor permeation. The decreasing recorded values in τ indicate a considerable lowering in the feed temperature at the T_fm_ compared to the temperature at the T_f_ on the hot-feed side. According to the obtained τ outcomes, TP has a dramatic effect on AGMD productivity (P_r_) compared to CP. Also, the negative TP impact was more obvious at high feed temperatures due to increasing vapor permeating the membrane. Therefore, it is concluded that TP is mainly responsible for a reduction in the increasing percentage of process productivity (P_r_) by 44%, 22%, and 14%. The obtained results are in good agreement with those of Curcio & Drioli [12] and Lawson & Lloyd [35]. As stated by Abu-Zeid et al. [50] and Alkhudhiri & Hilal [51], the thermal boundary layer (low τ value) is deemed a prime factor in restricting vapor mass transfer. For example, at a low T_f_ of 50 °C and high τ value, a small difference (∆T_f-fm_) in temperature between the bulk (T_f_) and interface (T_fm_) were 0.95 °C and 2.33 °C, respectively. Correspondingly, at a high T_f_ of 80 °C and low τ value, a large difference (∆T_f-fm_) in temperature between the bulk (T_f_) and interface (T_fm_) were 0.91 °C and 7.03 °C, respectively. Also, it was observed that the heat-transfer coefficient (h_f_) has a noticeable drop by 4.2%, 3.9%, and 3.5%, as shown in Figure 4c. The high decreasing percentage of h_f_ supports the conclusion that temperature polarization (TP) has a more effective influence on AGMD productivity (P_r_) than concentration polarization (CP). This suggests that differences in temperature across the membrane play a more significant role in affecting the overall process efficiency. The results of one-way ANOVA are listed in Table 4 and Figure 4a–c for P_r_, τ, and h_f_. From the one-way ANOVA table, it can be shown that the contribution of the feed temperature (T_f_) parameter is the most significant for determining that the process P_r_ and h_f_ (*p* < 0.001 ***) is dissimilar to τ, which was non-significant (*p* > 0.05). Also, the high F-ratios presented in the table support this result.

**Figure 5:**
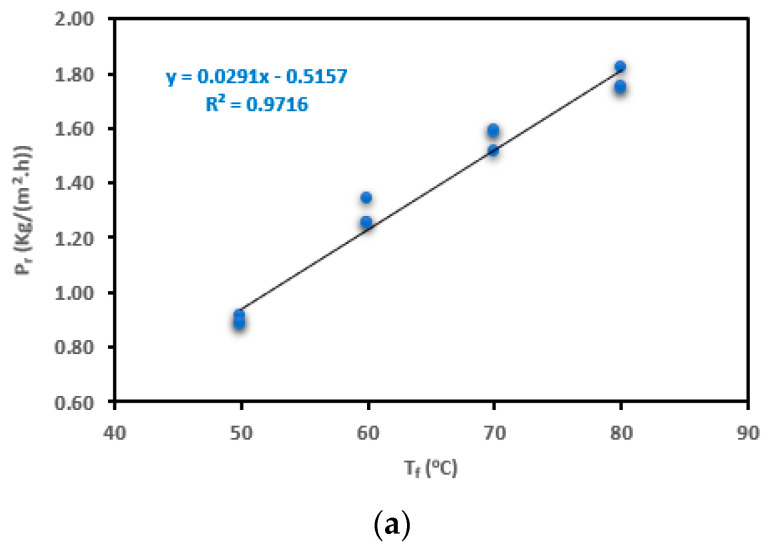

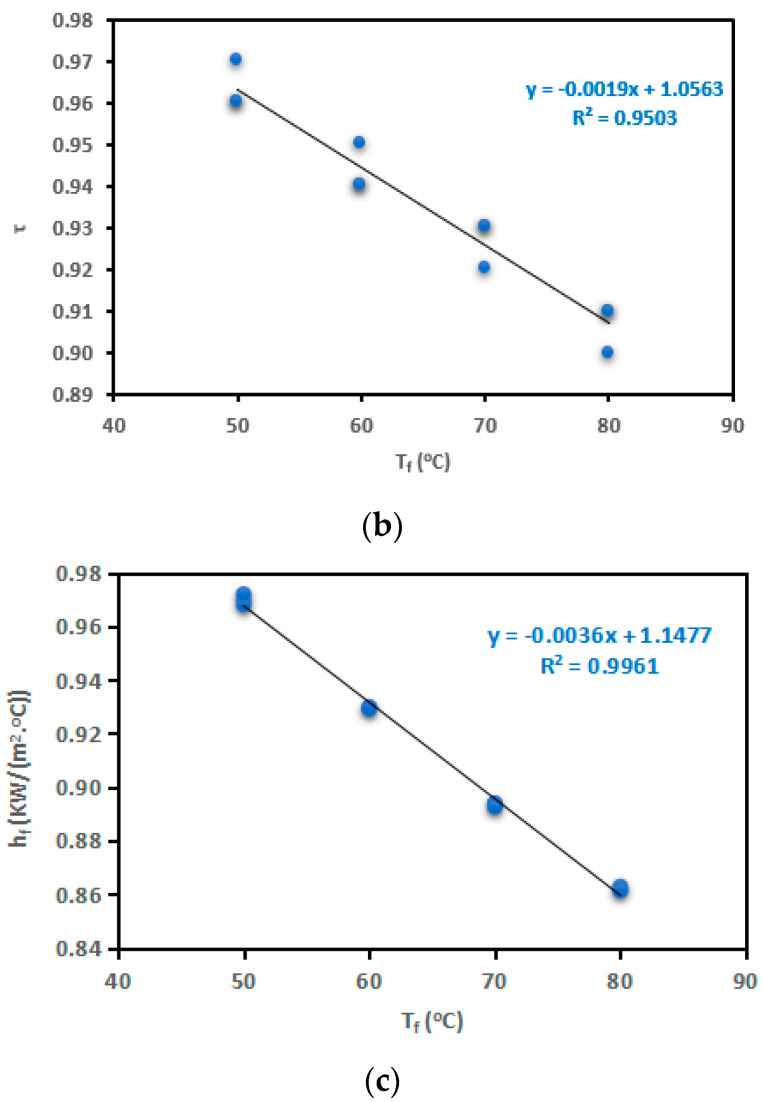
The correlation between feed temperature (T_f_) and (**a**) P_r_, (**b**) τ, and (**c**) h_f_ for the SP-AGMD system.

A strong direct linear relationship (correlation coefficient > 0.90) is observed between the variable feed temperature (T_f_) and both productivity (P_r_) and heat-transfer coefficient (h_f_). This implies that as T_f_ increases, P_r_ and h_f_ also tend to increase, and this relationship is highly consistent, as shown in Table 5. There is a weak linear relationship between T_f_ and τ. This indicates that as T_f_ increases, τ tends to decrease. The strength of the relationship suggests that temperature changes influence these variables. There is a non-significant negative linear relationship between T_f_ and τ. This means that changes in T_f_ do not strongly predict changes in τ. There are non-significant negative linear relationships between T_f_ and h_f_. This indicates that changes in T_f_ are not strongly linked to changes in h_f_, and these relationships might not be reliable. Results reveal strong positive relationships between T_f_ and P_r_ and h_f_, suggesting that, as temperature increases, these variables tend to increase significantly, as shown in Figure 5. There are also negative relationships between T_f_ and τ, indicating that changes in temperature correlate with a decrease in these variables. However, linear regression analysis shows that the relationships between T_f_, τ, and h_f_, are not statistically significant.

### 5.2. Effect of Temperature Polarization and Concentration Polarization on the Productivity at Different Feed Flow Rates (M_f_)

**Figure 6:**
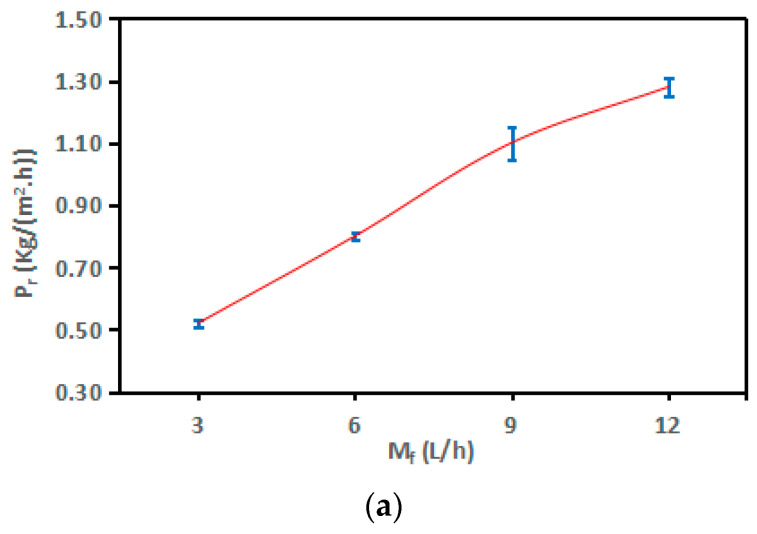

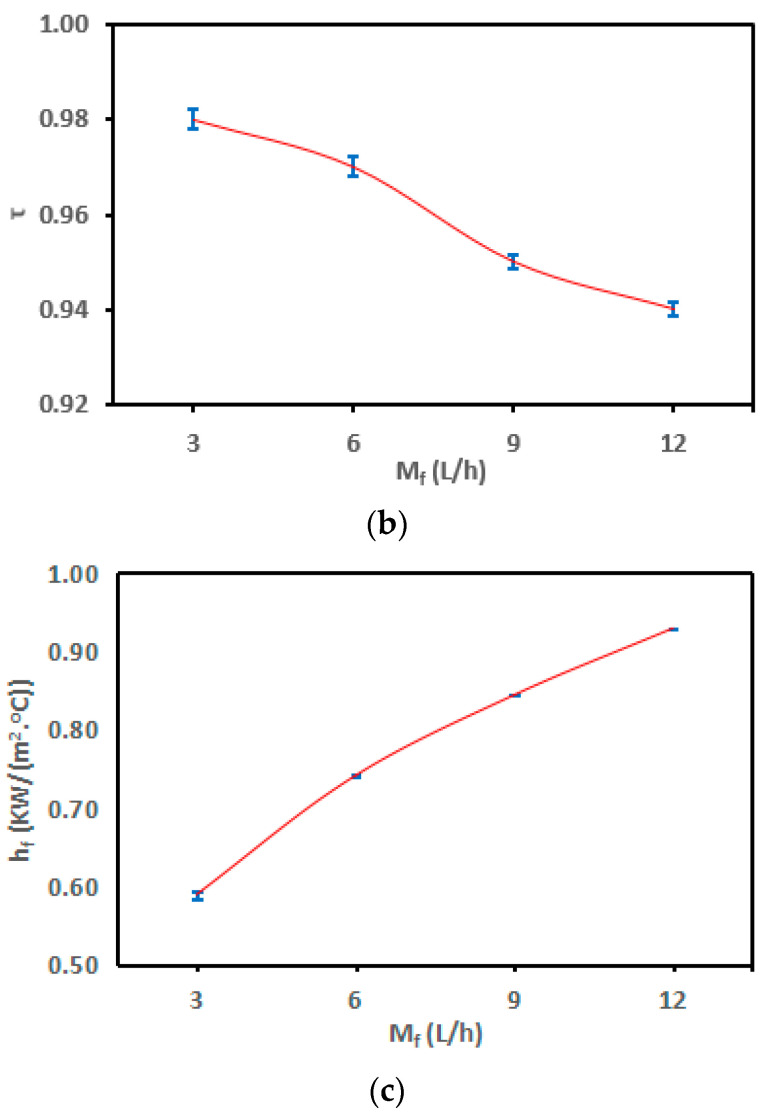
Change in the (**a**) P_r_, (**b**) τ, and (**c**) h_f_ for the SP-AGMD system at different flow rates (M_f_). With standard errors.

The experiments were conducted using flow rates of 3, 6, 9, and 12 L/h. These flow rates corresponded to feed velocities of 0.014, 0.028, 0.041, and 0.055 m/s, respectively. The experiments were performed at a feed temperature of 60 °C, a cooling temperature of 20 °C, and a concentration of 0.5%. An impressive increase of 146% in productivity (P_r_) was achieved, going from 0.52 to 1.28 kg/(m^2^·h) as the flow rates raised from 3 L/h to 12 L/h in increments of 3 L/h, as displayed in Figure 6a. These experimental findings aligned with the results by Zhang et al. [52] and Duong et al. [53].

Referring to the temperature polarization coefficient (τ), it is evident from Figure 6b that the τ of the AGMD module decreased by 1%, 2%, and 1%. As previously indicated in the context of feed temperature (T_f_), the reduction in flow rate (M_f_) significantly mitigated the adverse effects of concentration polarization (CP) by decreasing the thickness of concentration boundary layers, leading to a more pronounced difference in trans-membrane temperature [54]. Concerning thermal boundary layers, as explained by Xu et al. [55], the thickness of these layers remained relatively stable under the tested laminar flow conditions (Reynolds number (Re) < 2100), which resulted in a significant decrease in the observed increase in AGMD productivity (P_r_) percentages, specifically: 54%, 38%, and 16%. At a flow rate (M_f_) of 3 L/h, the temperature polarization coefficient (τ) exhibited a high value of 0.98 °C, accompanied by a small trans-membrane temperature difference (T_f_ − T_fm_) of 2.59 °C. In contrast, at a flow rate (M_f_) of 12 L/h, the τ value was low at 0.94 °C, while the trans-membrane temperature difference (T_f_ − T_fm_) was larger at 3.68 °C.

Figure 6c exhibited notable increments of 26%, 14%, and 10% in the heat-transfer coefficient (h_f_). Given that the mass and heat transfer processes transpired simultaneously within the AGMD module [56], the enhancement in the heat-transfer coefficient (h_f_) would consequently be mirrored by analogous increases in the mass-transfer coefficient (K_f_).

Observations drawn from the experimental results showed that elevating T_f_ and M_f_ yielded remarkable enhancements in the driving force for the permeation and productivity (P_r_) of the AGMD process, with approximate increments of 101% and 146%, respectively. Termpiyakul et al. [25] stated that an increase in AGMD productivity (P_r_) led to negative impacts on TP and CP. The experimental findings further highlighted that, in the context of flow rate (M_f_), the computed average reduction percentage of τ was 4%. In the scenario of feed temperature (T_f_), these figures showed an approximately 6% reduction. Consequently, distinct from the effect of feed temperature (T_f_), the current laminar flow regime and the elevated heat-transfer coefficient (h_f_) worked to mitigate the negative effects of TP and CP. Similar findings have been reported [24,57].

The one-way ANOVA results are given for P_r_, τ, and h_f_ in Table 6 and Figure 6a–c. Due to *p*-values < 0.05, the results of ANOVA showed that the flow rate (M_f_) induced highly significant differences on P_r_, h_f,_ (*p* < 0.001 ***) and a significant difference on τ (*p* < 0.05 *). Also, large *F*-ratios corresponding to the flow rate (M_f_) are considered the most influential parameter.

**Figure 7:**
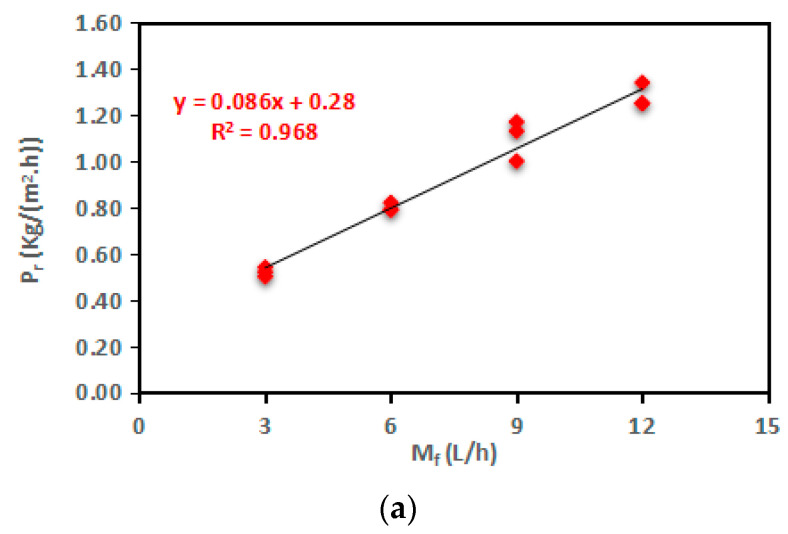

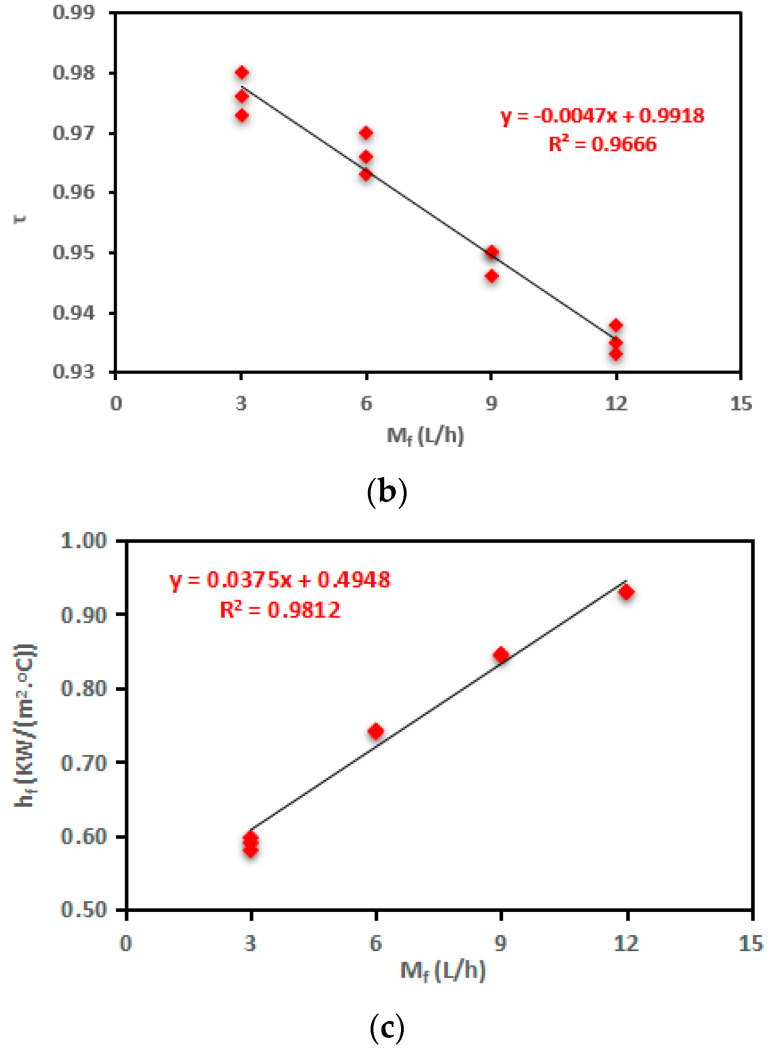
The correlation between feed flow rate (M_f_) and (**a**) P_r_, (**b**) τ, and (**c**) h_f_ for the SP-AGMD system.

The outcomes of Spearman’s correlation analysis, outlined in both Table 7 and Figure 7a–c, reveal a highly robust positive linear correlation between M_f_ and P_r_, as depicted in Figure 7a–c, owing to correlation coefficients (r) exceeding 0.90. Simultaneously, they indicate a relatively weak adverse linear correlation between M_f_ and τ, as shown in Figure 7b. They are following the execution of a simple linear regression and the application of linear regression trendlines between M_f_ and each of τ and h_f_. Similarly, the correlation between M_f_ and τ displays a weak negative linear relationship that is also considered non-significant.

### 5.3. Effect of Temperature Polarization and Concentration Polarization on the Productivity at Different Feed Salt Concentrations (C_f_)

**Figure 8:**
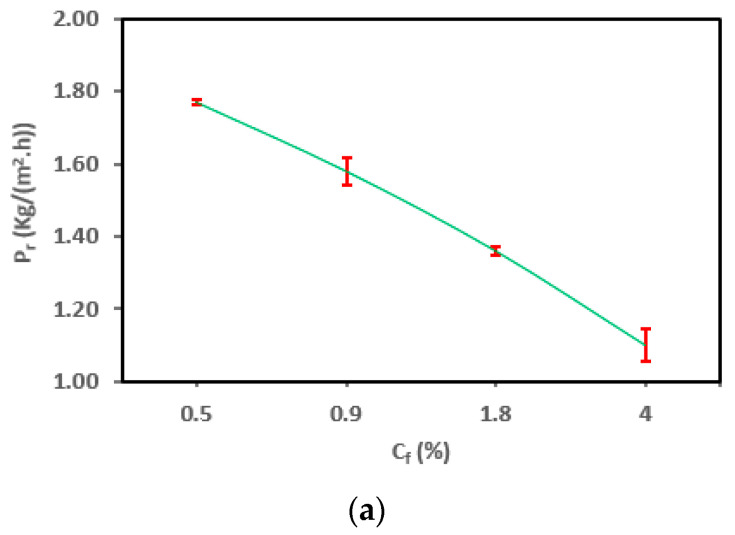

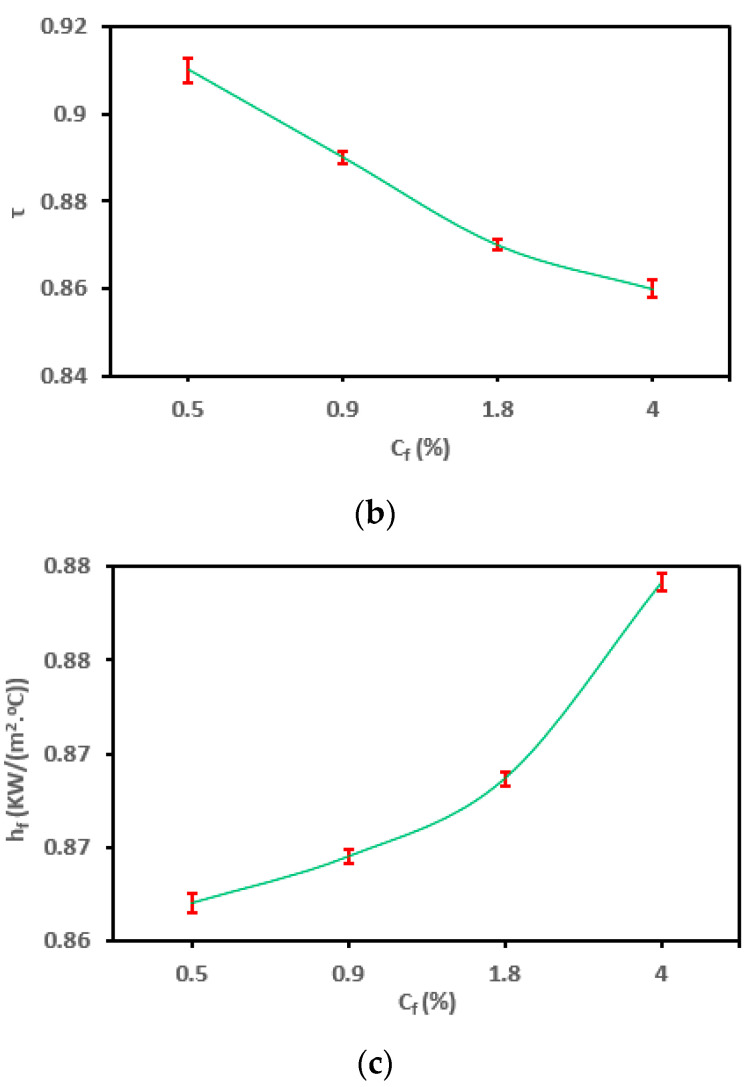
Change in the (**a**) P_r_, (**b**) τ, and (**c**) h_f_ for the SP-AGMD system at different feed salt concentrations (C_f_), with standard errors.

The productivity of the module changes is based on varying salt concentration (C_f_), as shown in Figure 8a. The experiment was conducted under a cooling temperature of 20 °C, a feed temperature of 80 °C, and a flow rate of 12 L/h. As illustrated in Figure 8a, there was a reduction of 37.57% in productivity, decreasing from 1.73 to 1.08 kg/(m^2^·h), as the salt concentration (C_f_) gradually increased from 0.5% to 4%. These findings align entirely with the outcomes reported in another study [51]. The decline in productivity can be attributed to a decrease in vapor pressure difference and water activity, which is linked to a low Prandtl number (P_r_) [35,51]. Furthermore, the detrimental effects of thermal polarization (TP) and concentration polarization (CP) also contribute to this phenomenon [51].

Figure 8b shows reductions of 0.88%, 1.11%, and 2.14% in the τ value. Higher salt concentrations (C_f_) resulted in a decrease in the vapor pressure of the feed solution. This led to a reduction in the effective driving force for vapor transport across the membrane. Consequently, temperature polarization (TP) can become more pronounced. With higher salt concentrations, the concentration of solutes near the membrane surface increases. This concentration polarization (CP) can create a concentration gradient that, in turn, affects the vapor pressure gradient and contributes to temperature polarization (TP).

As depicted in Figure 8c, there was an enhancement of the heat-transfer coefficient (h_f_) with increasing salt concentration (C_f_). The presence of dissolved salts can alter the heat-transfer characteristics of the feed solution. This can impact the rate at which heat is conducted through the feed solution layer, further influencing the temperature polarization gradient. The modest increments in h_f_ by 0.3%, 0.5%, and 1.2% can be predominantly attributed to the decreased heat-transfer requirements due to lower productivity (P_r_) at higher salt concentrations (C_f_) and smaller ΔT_f-fm_. This trend aligns with the flow-rate data but contradicts observations related to feed temperature. Notably, the rate of salt rejection surpassed 99% across various operating conditions, consistent with findings from a similar study [58,59].

The outcomes of one-way ANOVA for P_r_, τ, and h_f_ are listed in Table 8 and Figure 8a–c. As presented in Table 8, the salt concentration (C_f_) contribution is the most significant for estimating the P_r_, h_f_ (*p* < 0.001 ***), and τ (*p* < 0.01 **). Also, the calculated large *F*-ratios listed in the table emphasize this outcome. The slight influence of CP suggests that the AGMD module could potentially be employed for treating highly saline streams, in line with findings reported by Duong et al. [60].

**Figure 9:**
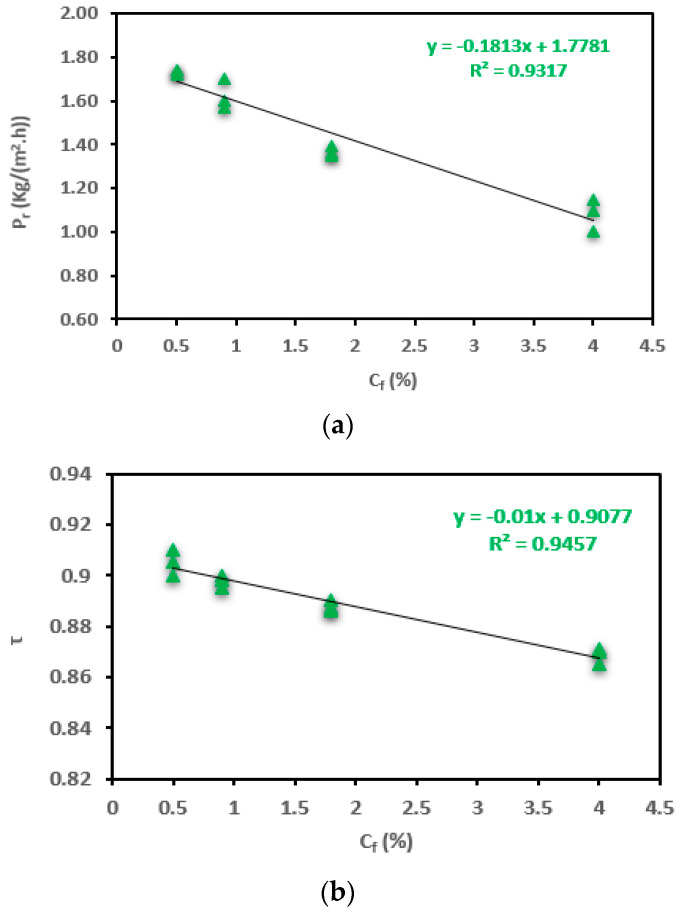

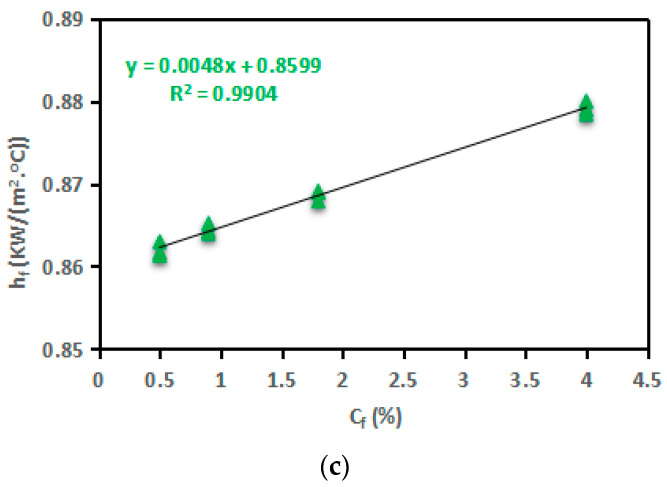
The correlation between feed salt concentration (C_f_) and (**a**) P_r_, (**b**) τ, and (**c**) h_f_ for the SP-AGMD system.

Table 9 and Figure 9a–c display the results of Spearman’s correlation analysis. The correlation coefficients (r) demonstrated strong and weak negative linear relationships between C_f_ and each of P_r_ and τ, respectively (Figure 9a,b), while showing a very strong positive linear relationship between C_f_ and h_f_ (Figure 9c). Simple linear regression and linear regression trendlines proceeded between C_f_ and h_f_. Accordingly, there was a significant positive linear relationship between C_f_ and h_f_.

In comparison to salt concentration and feed temperature, variations in flow rate mitigated the negative impact of temperature polarization (TP) and enhanced productivity (P_r_). The recorded average reduction percentages of the temperature polarization coefficient (τ) were 4.2%, 6%, and 4% for M_f_, T_f_, and C_f_, respectively.

### 5.4. Performance of the SP-AGMD System and Solar Collector during Winter and Summer

**Figure 10:**
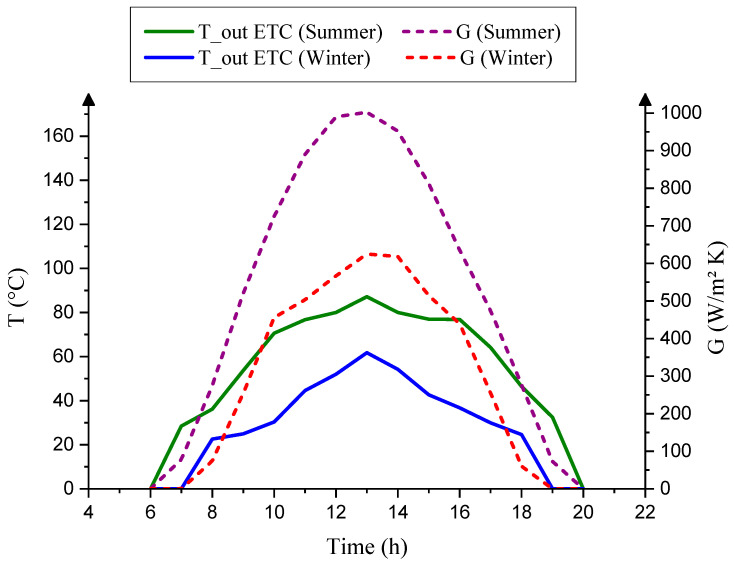
Temperature of ETC and global radiation in winter and summer.

Figure 10 illustrates the changes in global radiation (G) impacting the outlet temperature collector under varying weather conditions. Notably, the outlet temperature reached 61 °C during January, with a solar radiation of 625 W/m^2^·K. In contrast, the temperature increased to 85 °C in August, coinciding with a solar radiation of 1002 W/m^2^·K. This observation highlights a significant alteration in the outlet temperatures of the ETC, indicating a 39% variation between winter and summer days.

**Figure 11:**
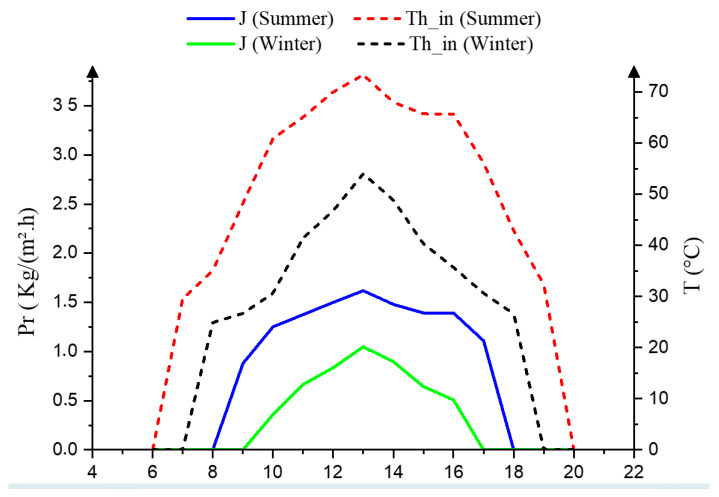
Temperature of ETC and water productivity of Port Said City weather.

Figure 11 displays the variations in AGMD unit productivity and inlet hot temperatures during both summer and winter daytime conditions. The AGMD system employed PVDF hollow-fiber membranes with a total internal membrane surface area of 0.36 m^2^, a pore size of 0.20 µm, a contact angle of 80.5°, a bubble point pressure of 0.11 MPa, and a porosity of 85%, all operating at a flow rate of 12 L/h. When the inlet hot temperatures of the AGMD unit are elevated to 54 °C and 73 °C, the corresponding productivity in January and August reach 1.05 and 1.62 kg/(m^2^·h), respectively. This observation underscores the significant impact of the AGMD’s inlet hot temperature on enhancing module productivity. Evidently, the percentage increase of 35% is discernible in the productivity between winter and summer days.

### 5.5. Photovoltaic (PV) Panels for the Solar-Driven AGMD System

**Figure 12:**
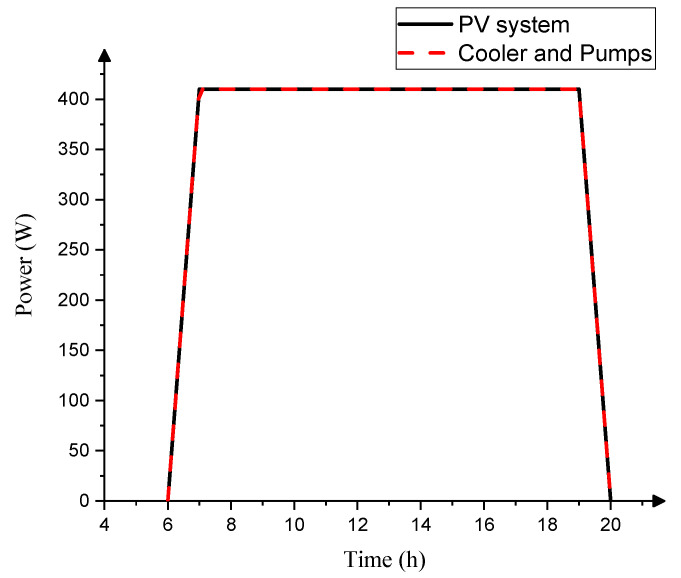
Comparison between the power of both the cooler and pumps with the PV system for the SP-AGMD system.

The operation of the AGMD process requires the electrical power of the circulation water pumps for the ETC and AGMD components. To minimize costs and leverage renewable energy sources, photovoltaic (PV) panels were employed in the solar-driven AGMD system. The required electrical power was determined and subsequently offset by the deployment of two PV panels. Each of these panels occupied an area of 1.6 m^2^ and was integrated with three batteries (12 V, 200 Ah), all analyzed using the TRNSYS program. Through this approach, it has become evident that the electric power consumed by the cooler and pumps aligns with the power generated by the PV system, amounting to 410 watts, as shown in Figure 12. It was found that there was a specific electricity demand of 0.4 kWh/m^3^.

## 6. Conclusions

To optimize cost efficiency, a solar AGMD system was analyzed by integrating solar collectors and photovoltaic (PV) panels using the TRNSYS program across different seasons and weather conditions. The system achieved a temperature of 85 °C with a solar radiation of 1002 W/m^2^·K during the summer season. By elevating the inlet hot temperature of the AGMD module to 73 °C, productivity increased to 1.62 kg/(m^2^·h). The corresponding electrical power was computed and substituted using two PV panels, each occupying an area of 1.6 m^2^, generating 300 W power, and integrated with three batteries (12 V, 200 Ah). Consequently, the evacuated tube collector (ETC) facilitated water heating across an area of 0.25 m^2^, and the PV system’s power effectively replaced the energy required by the cooler and circulation water pumps in the solar AGMD system. Furthermore, Spearman’s correlation analysis showed remarkable results, revealing a highly robust positive linear relationship between M_f_ and parameters such as P_r_ and h_f_. However, it exhibited a weaker negative linear relationship between M_f_ and τ. The subsequent application of simple linear regression and linear regression trendlines to M_f_ and the respective variable τ indicates that no significant negative linear relationship emerged between M_f_ and τ. When examining stable conditions characterized by C_f_ at 0.5% and Tc at 20 °C, notable trends were observed. By increasing T_f_ (at M_f_ of 12 L/h) and M_f_ (at T_f_ of 60 °C), P_r_ increased by 99% and 146%, respectively. However, with the elevation of C_f_ (at M_f_ of 12 L/h and T_f_ of 80 °C), P_r_ experienced a decline of approximately 37.57%. This trend emphasized the complex interplay of parameters in AGMD processes. The calculated average percentage decrease of τ amounted to 4.2%, 6%, and 4%, revealing the diminishing influence of temperature polarization under varying conditions. This integrated AGMD system, which utilizes renewable energy resources for desalination, holds promise for broader technological applications. Such solar-based projects present compelling economic benefits and profitability.

## Figures and Tables

**Table 1:** The parameters of the Evacuated Tube Collector (ETC).

Parameter	Value
**Evacuated Tube Collector (ETC)**	
Grid measurement (length, height) (mm)	500 × 500
Aperture area (m^2^)	2.5
Efficient solar absorption area (m^2^)	2.44
Fluid capacity in copper pipe (L)	1.82
Working pressure maximum (bar)	6

**Table 2:** The specifications of the PV panel.

Specifications	Value
PV panel	
Power (kW)	0.3
Module area (m^2^)	1.6
Voltage (V)	38.9
Current (A)	9.31
Inverter	
High fractional condition of charge limit	1.0
Regulator efficiency	0.78
Battery	
Tolerance for iterative calculations	16.7
Charging efficiency (A)	0.8

**Table 3:** The specifications of the circulation pump, evacuated tube, and heat exchanger employed in the proposed system.

Specification	Value
Circulation pump	
Power (kW)	0.09
Voltage (V)	220
Frequency (Hz)	50
Speed (rpm)	2800
Max. flow (L/min)	25
Highest lift (m)	10
Current (A)	0.95
Max. head (m)	8
Evacuated tube	
Power (kW)	1.5
Voltage (V)	220
Frequency (Hz)	50
Highest temperature (°C)	95
Heat exchanger	
Power (kW)	0.23
Voltage (V)	220
Frequency (Hz)	50
Max. flow (L/min)	15
Highest lift (m)	10
Refrigerating capacity (kW)	0.550–0.275
Max. head (m)	3

**Table 4:** One-way analysis of variance (ANOVA) results for P_r_, τ, and h_f_ at T_f_.

Variable (Parameter)	ANOVA		
	df	*F*-Ratio	*p*-Value
P_r_	3	227.8	<0.0001 *
τ	3	2.92	>0.999 ns
h_f_	3	4547.9	<0.0001 **

* significant at *p* < 0.05; ** highly significant at *p* < 0.01 and <0.001; ns—non-significant at *p* > 0.05.

**Table 5:** Spearman’s correlation analysis results for P_r_, τ, and h_f_ at T_f_.

Variables		Correlations			
		T_f_	P_r_	τ	h_f_
P_r_	r	0.973	---	−0.625 *	−0.921 **
	Sig. (2-tailed)	<0.0001 ***		0.030	0.000
τ	r	−0.70	−0.63	---	0.674 *
	Sig. (2-tailed)	0.012 *	0.030 *		0.016
h_f_	r	−0.97	−0.921	0.67	---
	Sig. (2-tailed)	<0.0001 ***	<0.0001 ***	0.016 *	

* significant at *p* < 0.05, **, *** highly significant at *p* < 0.01 and <0.001; non-significant at *p* > 0.05.

**Table 6:** One-way analysis of variance (ANOVA) results for P_r_, τ, and h_f_ at M_f_.

Variables	ANOVA		
	df	*F*-Ratio	*p*-Value
P_r_	3	119	<0.0001 **
τ	3	4.44	<0.05 *
h_f_	3	3364.8	<0.0001 **

* significant at *p* < 0.05, ** highly significant at *p* < 0.01 and <0.001; non-significant at *p* > 0.05.

**Table 7:** Spearman’s correlation analysis results for P_r_ and τ at M_f_.

Variables		Correlations			
		M_f_	P_r_	τ	h_f_
P_r_	r	0.975			
	Sig. (2-tailed)	<0.0001 ***			
τ	r	−0.80	−0.75		
	Sig. (2-tailed)	0.002 **	0.005 **		
h_f_	r	0.972	0.933	−0.74	
	Sig. (2-tailed)	<0.0001 ***	<0.0001 ***	0.006 **	

**, *** highly significant at *p* < 0.01 and <0.001; non-significant at *p* > 0.05.

**Table 8:** One-way analysis of variance (ANOVA) results for P_r_, τ, and h_f_ at C_f_.

Variable (Parameter)	ANOVA		
	df	*F*-Ratio	*p*-Value
P_r_	3	72.4	<0.0001 ***
τ	3	8.4	<0.01 **
h_f_	3	306.6	<0.0001 ***

**, *** highly significant at *p* < 0.01 and <0.001; non-significant at *p* > 0.05.

**Table 9:** Spearman’s correlation analysis results for P_r_, τ, and h_f_ at C_f_.

Variables		Correlations			
		C_f_	P_r_	τ	h_f_
P_r_	r	−0.973			
	Sig. (2-tailed)	<0.0001 ***			
τ	r	−0.873	0.89		
	Sig. (2-tailed)	<0.0001 ***	<0.0001 ***		
h_f_	r	0.972	−0.91	−0.781	
	Sig. (2-tailed)	<0.0001 ***	<0.0001 ***	0.003 **	

**, *** highly significant at *p* < 0.01 and <0.001; non-significant at *p* > 0.05.

## Data Availability

Data will be made available upon request.

## Nomenclature

A	Effective membrane area, m^2^
AGMD	Air-gap membrane distillation
C_f_	Bulk concentration at hot-feed flow, %
C_fm_	Interface concentration on the membrane surface on the hot-feed side, %
C_w_	Distilled water salinity, %
C_P_	Liquid heat capacity at constant pressure, J/kg·°C
CP	Concentration polarization
d_i_	Tube/hollow-fiber membrane internal diameter, m
D_AB_	Diffusivity coefficient of water vapor (A) relative to air (B), m^2^/s
h_f_	Heat-transfer coefficient through the tube-side thermal boundary layer, W/(m^2^·°C)
K_f_	Solute diffusive mass transfer coefficient through the boundary layers, W/(m^2^·°C)
K	Liquid thermal conductivity, W/(m·°C)
M_f_	Flow rate
n	Number of permeating species
P_r_	Productivity, kg/(m^2^·h)
Pr	Prandtl number
Q_f_	Effective heat transfer over the thermal feed side boundary layer, kJ/h
Q_v_	Rate of latent heat, kJ/h
Re	Reynolds number
Sh	Sherwood number
Sc	Schmidt number
t	Operation time, h
T_f_	Hot bulk feed temperature, °C
T_fm_	Interface temperature, °C
T_c_	Coolant temperature, °C
TP	Temperature polarization
∆T_f-fm_	Difference in temperature between bulk (T_f_) and interface membrane surface (T_fm_) at the hot-feed side, °C
∆H_V_	Latent heat of evaporation, kJ/kg
v	Linear velocity, m/s
Nu	Nusselt number
W_r_	Distilled water volume, kg
**Greek Symbols**	
γ	Concentration polarization coefficient
τ	Temperature polarization coefficient
ρ	Bulk liquid density, kg/m^3^
µ	Bulk liquid dynamic viscosity, kg/(m·s)
